# Direct-fed *Enterococcus faecium* plus bacteriophages as substitutes for pharmacological zinc oxide in weanling pigs: effects on diarrheal score and growth

**DOI:** 10.5713/ab.22.0262

**Published:** 2022-10-11

**Authors:** Sang-Hyon Oh, Jae-Cheol Jang, Chul Young Lee, Jeong Hee Han, Byung-Chul Park

**Affiliations:** 1 Department of Animal Resources Technology, Gyeongsang National University, Jinju 52725, Korea; 2 College of Veterinary Medicine and Institute of Veterinary Science, Kangwon National University, Chuncheon 24341, Korea; 3 Graduate School of International Agricultural Technology and Institutes of Green Bio Science and Technology, Seoul National University, Pyeongchang 25354, Korea

**Keywords:** Bacteriophage, Diarrhea, *Enterococcus*, Growth Performance, Weanling Pig, Zinc Oxide

## Abstract

**Objective:**

Effects of direct-fed *Enterococcus faecium* plus bacteriophages (EF-BP) were investigated as potential substitutes for pharmacological ZnO for weanling pigs.

**Methods:**

Dietary treatments were supplementations to a basal diet with none (NC), 3,000-ppm ZnO (PC), 1×10^10^ colony-forming units of *E. faecium* plus 1×10^8^ plaque-forming units (PFU) of anti-*Salmonella typhimurium* bacteriophages (ST) or 1×10^6^ PFU of each of anti-enterotoxigenic *Escherichia coli* K88 (F4)-, K99 (F5)-, and F18-type bacteriophages (EC) per kg diet. In Exp 1, twenty-eight 21-day-old crossbred weanling pigs were individually fed one of the experimental diets for 14 days and euthanized for histological examination on intestinal mucosal morphology. In Exp 2, 128 crossbred weanling pigs aged 24 days were group-fed the same experimental diets in 16 pens of 8 piglets on a farm with a high incidence of post-weaning diarrhea.

**Results:**

None of the diarrheal score or fecal consistency score (FCS), average daily gain (ADG), gain: feed ratio, structural variables of the intestinal villus, and goblet cell density, differed between the EF-BP (ST+EC) and NC groups, between EF-BP and PC, or between ST and EC, with the exception of greater gain: feed for EF-BP than for PC (p<0.05) during days 7 to 14 (Exp 1). In Exp 2, ADG was less for EF-BP vs PC during days 0 to 7 and greater for EF-BP vs NC during days 7 to 14. FCS peaked on day 7 and declined by day 14. Moreover, FCS was less for EF-BP vs NC, did not differ between EF-BP and PC, and tended to be greater for ST vs EC (p = 0.099). Collectively, EF-BP was comparable to or slightly less effective than PC in alleviating diarrhea and growth check of the weanling pigs, with ST almost as effective as PC, when they were group-fed.

**Conclusion:**

The *E. faecium*-bacteriophage recipe, especially *E. faecium*-anti-*S. typhimurium*, is promising as a potential substitute for pharmacological ZnO.

## INTRODUCTION

Weaning of the piglets brings about enormous disturbances in their digestive function and health resulting in a transient growth check [1–3]. The digestive enzyme activity is reduced after weaning concordant with a damage of the intestinal villus structure resulting from the sudden change of the diet from mother’s milk to a solid feed. Furthermore, postweaning piglets frequently suffer from diarrhea caused mostly by *Salmonella typhimurium* or enterotoxigenic *Escherichia coli* (ETEC) K88 (F4) or F18 and less frequently by K99 (F5) or F41 [4–6]. The post-weaning diarrhea (PWD) was commonly prevented or alleviated by supplementing the commercial nursery diet with antibiotics, but such practice has been banned in the EU since 2006 [Regulation (EC) No 1831/2003] followed by Korea, USA and many other countries [7,8].

Zinc oxide (ZnO) was or is currently added to the weaner diet in many countries to a 2,000- to 4,000-ppm ‘pharmacological’ concentration, at which it has bactericidal, immunomodulatory, and other actions, including enhancement of digestive function, resulting in improvement of growth performance of the piglets [9,10]. However, dietary supplementation of ZnO has been limited to 150 ppm due to concerns about environmental pollution in the EU (Regulation [EC] No 1334/2003), which led to adoption or consideration of a similar regulation by a number of non-European countries [9,10], because dietary ZnO, like many other mineral supplements, is mostly excreted unabsorbed in feces [11,12]. Many mineral workers have hence studied the feasibility of replacing pharmacological ZnO with a low supplemental level of nano ZnO or a porous or coated form of ZnO which was designed to increase the bioavailability of the mineral particle [10]. Such efforts, however, were only partially successful, in that the nano or processed form of ZnO exhibited greater effects on alleviation of the porcine PWD and/or growth check than native ZnO as expected, but in no case was any of the former supplemented at 100 to 200 ppm unequivocally as effective as pharmacological ZnO. It is, therefore, necessary to search for alternative supplements substitutable for pharmacological ZnO.

Lactic acid bacteria, including the genera *Lactobacillus*, *Bifidobacterium*, and *Enterococcus*, have been used as direct-fed microbials for growing pigs for the past few decades [6,8,13]. Direct-fed *E. faecium*, which was used as a probiotic agent in the present study, has been shown to have beneficial effects on digestive function of growing pigs including the weaner [7,14,15]. However, the effects of direct-fed *E. faecium* on diarrheal score and growth performance, which, like those of many other probiotics, are often marginal or inconsistent depending on the supplementary concentration and duration of the probiotic, developmental or physiological stage of the subject, experimental conditions, and others. Moreover, effects of direct-fed *E. faecium* are increased ordinarily when *E. faecium* is provided with other microbes [6,8,13,15–19]. To our knowledge, however, no result has been reported so far regarding the effectiveness of the microbial recipe of *E. faecium* plus anti-*Salmonella* or anti-ETEC bacteriophages, which was used as the dietary treatment for the control of porcine PWD in the present study.

Bacteriophages have lately received renewed interests as direct-fed microbials for weaner pigs [20]. Gebru et al [21] and the present group [22,23] have reported that direct-fed bacteriophages lytic to *Salmonella enterica* and ETEC K99 and/or K88, respectively, alleviated the clinical symptoms including the decreased growth performance of the weanling pigs challenged with the pathogen(s) targeted by the direct-fed microphage(s) in the respective studies. Furthermore, Lee et al [24] and Hosseindoust et al [25] have reported that a cocktail of direct-fed bacteriophages targeted to several bacterial types, including *Salmonella*, ETEC, *Staphylococcus aureus*, and *Clostridium*, was as effective as pharmacological ZnO in reducing the diarrheal score and enhancing growth performance of intact weaner pigs. It is not known, however, how much of the observed effects were attributable to each bacteriophagic agent.

The present study was initiated to investigate the effects of direct-fed *E. faecium* plus anti-*S. typhimurium* bacteriophages or a mixture of anti-ETEC F4-, F5-, and F18-type bacteriophages on growth performance, diarrheal score, and intestinal mucosal morphology of weanling pigs, with an aim to find a feasibility of replacing pharmacological ZnO with the *E. faecium*-bacteriophage recipe. Additionally examined in the present study was how the piglets would respond to the dietary recipe when they are group-fed like the commercial pigs compared to when individually fed as under experimental settings.

## MATERIALS AND METHODS

All experimental protocols involving animals of the present study were approved by the Institutional Animal Care and Use Committee (IACUC) of Gyeongsang National University (GNU-220519-P0046).

### Experiment 1

Twenty-eight castrated piglets born to Duroc-sired (Landrace ×Yorkshire) dams were housed in as many pens with a 0.45-m^2^ floor space each installed with a feeder and a nipple waterer and randomly allotted to four dietary treatments, with seven animals (pens) per treatment, upon weaning at 21 days of age on an ordinary commercial farm with regard to the incidence of PWD [26,27]. The animals were fed *ad libitum* a typical nursery diet (Table 1) supplemented with (Table 2) none (negative control; NC), 3,000-ppm ZnO (positive control; PC), or 1×10^10^ colony-forming units of *Enterococcus faecium* plus bacteriophages (EF-BP) lytic to *S. typhimurium* [ST; 1×10^8^ plaque-forming units (PFU)/kg diet] or lytic to each of ETEC F4, F5, and F18 types (EC; 1×10^6^ PFU each/kg). The basal diet was formulated to meet the nutrient composition for the early nursery pig recommended by NIAS [28]. The direct-fed *E. faecium* and bacteriophages (CTCBIO, Inc., Seoul, Korea) used in the present study were isolated from infant feces and porcine feces collected at several commercial farms [23–25], respectively.

Body weight was measured on days 0, 7, and 14 of the experiment, when the fecal consistency was scored simultaneously as previously described [26,27]: 1, normal firm feces; 2, soft feces; 3, diarrhea. Feed intake was measured on days 7 and 14. All animals were euthanized by electric stunning upon termination of the 14-day feeding trial, after which intestinal tissue samples were excised, fixed in a 10% neutral formaldehyde buffer, embedded in paraffin, mounted on the glass slide, and stained with hematoxylin and eosin, followed by microscopic examination, including determination of the villus height (VH), crypt depth (CD), and goblet cell density, also as described [22,26,27].

### Experiment 2

A total of 128 (Landrace×Yorkshire)×Duroc piglets were randomly allotted to sixteen 1.4 m×1.5 m pens, with four pens per dietary treatment and four female and four castrated piglets per pen, at weaning at 24 days of age on a second commercial farm where the incidence of PWD was high based on the farm record and visual inspection on the diarrheal status of the weanling pigs prior to the feeding trial. The animals received the 14-day feeding trial as in Exp 1, including the scoring/measurement of fecal consistency and growth performance, but the histological examination was not performed in Exp 2.

### Statistical analysis

All data were analyzed using the MIXED procedure of SAS (SAS/STAT Software for PC, Release 9.2; SAS Institute, Cary, NC, USA). The pen was the experimental unit in both Exp. The model included the dietary treatment or simply treatment as the main effect in all variables other than fecal consistency score (FCS). As for the analysis of overall effects of treatment, day, and treatment × day interaction of FCS, the model included the pen nested within treatment, day, treatment × day, day × pen in addition to treatment and the effects of the day and the day × treatment interaction were tested using the day × pen as the error term. The sex was included in the model as a random effect in Exp 2. Differences between EF-BP (ST+EC) and NC, between EF-BT and PC, and between ST and EC, which were tested by the pre-planned contrast, were judged significant when the corresponding p-value was 0.05 or less.

## RESULTS

### Experiment 1

Average daily gain (ADG) did not differ between the EF-BP and NC groups, between EF-BP and PC, or between ST and EC (Table 3) during any period of days 0 to 7, 7 to 14, and 0 to 14 of the feeding trial in Exp 1 in which the piglets were individually fed. Average daily feed intake (ADFI) was greater for PC than for EF-BP during days 0 to 7, 7 to 14, and 0 to 14, with no difference between EF-BP and NC or between ST and EC during any period. In contrast, the gain:feed ratio was greater for EF-BT vs PC during days 7 to 14.

The diarrheal score or FCS did not differ between EF-BP and NC or PC or between ST and EC on any of days 0, 7, and 14. However, FCS tended to increase transiently between days 0 and 7 (1.14 and 1.43 for days 0 and 7, respectively; standard error of the mean [SEM] = 0.11), but neither the difference between the two days (p = 0.06) nor the day effect (p = 0.15) was significant. Overall, FCS did not differ between EF-BP and NC, between EF-BP and PC, or between ST and EC.

The VH, CD, and VH:CD ratio did not differ between EF-BP and NC or PC or between ST and EC in the duodenum, jejunum, or ileum (Table 4). The goblet cell density in the colon also did not differ between EF-BP and NC or PC or between ST and EC.

### Experiment 2

The rate of weight gain of the piglets for Exp 2, as a whole, was much less compared to that for Exp 1 during the feeding trial for the former in which the experimental animals were group-fed on a farm with a high incidence of PWD (Table 5). The ADG was less for EF-BP vs PC during days 0 to 7 and was greater for EF-BP vs NC during days 7 to 14, with no difference between ST and EC during any period. The ADFI was greater for EF-BP vs NC during days 7 to 14, otherwise no difference was detected between EF-BP vs NC or PC or between ST and EC during any other period. The gain:feed ratio did not differ between any two groups compared by the pre-planned contrast during any period.

FCS did not differ between EF-BP and NC or PC or between ST and EC on either day 0 or 7. On day 14, however, FCS was less for EF-BP than for NC, not being different between EF-BP and PC or between ST and EC. Overall, FCS was less for EF-BP vs NC and was not different between EF-BP and PC. Moreover, FCS peaked on day 7 and declined by day 14 to an intermediate level between those for days 0 and 7 (1.07, 1.70, and 1.32 for days 0, 7, and 14, respectively; SEM = 0.05; p<0.01). The temporal pattern in FCS was somewhat inconsistent among NC, PC, and EF-BP, but the treatment × day interaction was not significant (p = 0.36).

## DISCUSSION

Results of Exp 1 indicated that the EF-BP treatment is no different from NC or PC in its effects on the diarrheal score or weight gain of post-weaning piglets when they are individually penned. The greater gain:feed ratio for EF-BP vs PC during days 7 to 14 was noteworthy; however, this was more attributable to the increased ADFI by pharmacological ZnO as often observed in other studies [27,29] than to the effects EF-BP, because the ADG or gain:feed ratio was not different between EF-BP and NC. These results were similar to those of our previous studies [26,30] in which none of the growth enhancers examined, including antibiotics, direct-fed microbials, phytochemicals, and pharmacological ZnO exhibited any significant effect on growth performance or FCS when the weanling pigs were penned individually or in small groups. As such, these results are thus consistent with the notion that effects of the growth enhancers diminish under good environmental conditions like those of the experimental housing where the animals are less exposed to the pathogens and hence can maintain a better health status than under farm production conditions [2,3].

The lack of effects on the intestinal villus variables and the goblet cell density in Exp 1 was similar to the results of our previous studies [26,27] where even pharmacological ZnO exerted no effect on the villus morphology of weanling pigs. These results, however, are somewhat different from those of Lee et al [24] and Hosseindoust et al [25] where VH increased substantially in the jejunum and/or duodenum of weanling pigs in response to a 5-week dietary treatment with either pharmacological ZnO or a mixture of bacteriophages lytic to several bacterial types in post-weaning piglets. In our study, the structural variables of the villus did not change in response to a 2-week dietary treatment with pharmacological ZnO under either experimental [26] or on-farm [27] conditions, unless the piglets were challenged with an ETEC pathogen infecting the intestine [29]. It thus remains to be known if these different results had any relation to the differences in the microbial formula or the duration of the dietary treatment between the present and aforementioned [24,25] studies.

Exp 2 was performed on a farm where the incidence of PWD was high, on account of the fact that effects of a probiotic or antimicrobial agent are more pronounced on such a farm than on a cleaner farm or under experimental settings [2,3]. All four dietary groups of the present study, as expected, exhibited a greater increase in FCS (0.88) and depressed weight gains during days 0 to 7 compared to those of Exp 1 performed on a farm with a lower incidence of PWD which had exhibited FCS increases of 0.04 and 0.41 with 4- [26] and 34-piglet [27] pen sizes, respectively, for the NC group by the present scoring standard during the first seven days *postweaning* (0.15 increase in the present study). Furthermore, it was also evident that the piglets were recovering from the diarrheal stressor in all groups during days 7 to 14, which was indicated by the increases in ADG and gain: feed ratio as well as a decrease of FCS during this period in all treatments but PC. Regarding the effects of the treatments during this time-course of PWD, EF-BP was comparable to PC in reducing FCS and enhancing growth, but only PC was effective for alleviating the post-weaning growth check during the early acute phase (days 0 to 7) of PWD.

The ST tended to be more effective than EC in decreasing FCS in Exp 2, although the difference in FCS between the two treatments did not reach the 5% significance level (p = 0.099). Inasmuch as ST contained a 100-fold greater bacteriophage concentration than EC (1×10^8^ PFU vs 1×10^6^ PFU per ETEC type/kg diet), more studies are needed to figure out if the tendency had any relation to the microbial dose and, if so, to determine the optimum bacteriophage doses for both ST and EC. In this connection, Lee et al [24] and Hosseindoust et al [25] have reported that dietary supplementation of a cocktail of bacteriophages lytic to ETEC F4, F5, and F41 types, three *Salmonella* types including *typhimurium*, two *Clostridium perfringens* types, and *Staphylococcus aureus*, with a dose of 1×10^6^ PFU each/kg, were as effective as pharmacological ZnO in reducing the diarrheal score and/or enhancing growth performance of post-weaning piglets. It is hard to compare the effects of the microbial treatments in the present vs these studies because of the wide difference in the microbial formulas between the studies, yet the greater effects of the bacteriophage cocktail vs present EC relative to those of pharmacological ZnO are attributable to the bacteriophages included in the cocktail targeted to those bacterial types other than the ETEC.

Collectively, EF-BP was comparable to or slightly less effective than PC in alleviating PWD and growth check, with ST almost as effective as the latter, only in group-fed weanling pigs. In the individually-fed piglets, however, none of the treatments was effective for enhancing the performance or the integrity of the intestinal mucosal morphology. It is therefore concluded that the *E. faecium*-bacteriophage recipe, especially *E. faecium*-anti-*S. typhimurium*, is promising as a potential substitute for pharmacological ZnO.

## Figures and Tables

**Table 1:** Composition of the basal diet (as-fed basis)

Item	Content
Ingredient (%)	
Corn	33.17
Barley	8.00
Soybean meal	10.00
Dehulled soybean meal	10.00
Wheat bran	3.00
Limestone	0.30
Sweet whey	12.56
Lactose	4.20
Fish meal	5.00
Fermented soybean	4.15
Sucrose	3.00
Soy oil	3.00
Organic acids	0.70
Monocalcium phosphate	1.20
Salt	0.30
Vitamin premix^1)^	0.15
Mineral premix^2)^	0.20
Others^3)^	0.77
Total^4)^	99.70
Nutritional composition	
Digestible energy (Mcal/kg)	3.50
Crude protein (%)	18.50
Ether extract (%)	4.50
Lysine (%)	1.52

1) Provided per kg: 1,500 IU vitamin A, 2,000 IU vitamin D_3_, 65 IU vitamin E, 1.5 mg vitamin K, 1.0 mg thiamin, 6 mg riboflavin, 20 mg pantothenic acid, 25 mg niacin, 1.5 mg vitamin B_6_, 1 mg folic acid, 25 μg vitamin B_12_, 25 μg biotin, and 150 mg choline.

2) Provided per kg: 100 mg Zn (125-ppm ZnO), 160 mg Cu, 200 mg Fe, 40 mg Mn, 1 mg I, 0.15 mg Co, and 0.4 mg Se.

3) Provided per total weight: 0.1% choline-HCl, 0.347% L-lysine-HCl (78%), 0.150% DL-methionine (99%), 0.114% L-threonine (99%), 0.009% L-tryptophan, and 0.05% ethoxyquin.

4) Four experimental diets were supplemented with 3,000 mg ZnO, a combination of microbial agents, and 0 to 0.30% corn to make 100.00 for the sum of percentages. See Table 2 for details of supplementation of the dietary treatments.

**Table 2:** Supplementation of the experimental diets

Item	NC (Basal)	PC (ZnO)	EF-BP	

			ST	EC
Zinc oxide (%)		0.30		
EF^1)^ (%)			0.10	0.10
BP lytic to ST^2)^ (%)			0.10	
BP lytic to EC^3)^ (%)				0.10
Corn	0.30		0.10	0.10

NC, negative control; PC, positive control; EF, *Enterococcus faecium*; BP, bacteriophage; ST, *Salmonella typhimurium*; EC, *Escherichia coli*.

1) Contained 1×10^13^ colony-forming units (CFU)/kg (1×10^10^ CFU/kg diet).

2) Contained 1×10^11^ plaque-forming units (PFU)/kg (1×10^8^ PFU/kg diet).

3) A mixture of BP lytic to each of enterotoxigenic EC F4, F5, and F18 types (1×10^9^ PFU each; 1×10^6^ PFU each/kg diet).

**Table 3:** Effects of direct-fed probiotic *Enterococcus faecium* plus bacteriophages on growth performance and fecal consistency score of individually-fed weanling pigs (Experiment 1)^1)^

Item	NC (Basal)	PC (ZnO)	EF-BP		SEM	Contrast: p-value		
	
			ST	EC		EF-BP vs NC	EF-BP vs PC	ST vs EC
Body weight (kg)								
Day 0	5.23	5.78	5.54	5.52	0.27	0.38	0.47	0.96
Day 7	6.41	7.33	6.69	6.73	0.36	0.52	0.18	0.94
Day 14	8.83	9.60	8.93	9.12	0.42	0.69	0.28	0.75
ADG (g)								
Days 0–7	169	221	164	172	27	0.97	0.12	0.84
Days 7–14	345	325	321	343	23	0.64	0.80	0.47
Days 0–14	257	273	242	258	20	0.78	0.34	0.59
ADFI (g)								
Days 0–7	252	295	219	244	23	0.48	0.03	0.45
Days 7–14	606	685	573	575	37	0.49	0.02	0.98
Days 0–14	429	490	396	409	28	0.44	0.02	0.74
Gain:feed								
Days 0–7	0.664	0.750	0.730	0.702	0.070	0.55	0.70	0.78
Days 7–14	0.573	0.484	0.562	0.605	0.036	0.82	0.04	0.41
Days 0–14	0.602	0.566	0.611	0.633	0.037	0.66	0.23	0.67
Fecal consistency score^2)^								
Day 0	1.14	1.14	1.00	1.29	0.11	1.00	1.00	0.15
Day 7	1.29	1.43	1.43	1.57	0.11	0.49	0.82	0.69
Day 14	1.29	1.14	1.14	1.29	0.11	0.77	0.77	0.61
Overall^3)^	1.24	1.24	1.19	1.38	0.12	0.75	0.75	0.28

NC, negative control; PC, positive control; EF, *Enterococcus faecium*; BP, bacteriophage; ST, *Salmonella typhimurium*; EC, *Escherichia coli*; SEM, standard error of the mean; ADG, average daily gain; ADFI, average daily feed intake.

1) Weanling pigs were fed the basal diet supplemented with none (NC), 3,000 mg ZnO/kg (PC), or 1×10^10^ colony-forming units of EF plus BP (EF-BP) lytic to ST [1×10^8^ plaque-forming units (PFU)/kg] or lytic to each of enterotoxigenic EC F4, F5, and F18 types (EC; 1×10^6^ PFU each/kg) for 14 days. Data are means of seven piglets.

2) Fecal consistency was scored subjectively: 1, normal firm feces; 2, soft feces; 3, diarrhea.

3) The p-values for the day and day × treatment interaction were 0.15 and 0.99, respectively, with SEM = 0.11 and 0.21, respectively.

**Table 4:** Effects of direct-fed probiotic *Enterococcus faecium* plus bacteriophages on morphology of the intestine of individually-fed weanling pigs (Experiment 1)^1)^

Item	NC (Basal)	PC (ZnO)	EF-BP		SEM	Contrast: p-value		
	
			ST	EC		EF-BP vs NC	EF-BP vs PC	ST vs EC
Duodenum								
VH (μm)	293	284	263	268	14	0.12	0.27	0.80
CD (μm)	242	263	264	225	14	0.87	0.29	0.06
VH:CD	1.21	1.12	1.01	1.19	0.06	0.19	0.80	0.05
Jejunum								
VH	253	241	279	252	12	0.41	0.11	0.13
CD	201	184	211	201	9	0.64	0.07	0.46
VH:CD	1.27	1.33	1.33	1.26	0.08	0.80	0.68	0.56
Ileum								
VH	215	209	251	214	14	0.32	0.18	0.08
CD	161	165	177	167	12	0.47	0.67	0.56
VH:CD	1.42	1.23	1.41	1.31	0.07	0.50	0.15	0.32
Colon (cell density, cells/mm^2^)								
Goblet cell	955	1,012	928	915	51	0.58	0.16	0.86

NC, negative control; PC, positive control; EF, *Enterococcus faecium*; BP, bacteriophage; ST, *Salmonella typhimurium*; EC, *Escherichia coli*; SEM, standard error of the mean; VH, villus height; CD, crypt depth.

1) Weanling pigs were fed the basal diet supplemented with none (NC), 3,000 mg ZnO/kg (PC), or 1×10^10^ colony-forming units of EF plus BP (EF-BP) lytic to ST [1×10^8^ plaque-forming units (PFU)/kg] or lytic to each of enterotoxigenic EC F4, F5, and F18 types (EC; 1×10^6^ PFU each/kg) for 14 days. Data are means of seven piglets.

**Table 5:** Effects of probiotic *Enterococcus faecium* plus bacteriophages on growth performance and fecal consistency of group-fed weanling pigs (Experiment 2)^1)^

Item	NC(Basal)	PC (ZnO)	EF-BP		SEM	Contrast: p-value		
	
			ST	EC		EF-BP vs NC	EF-BP vs PC	ST vs EC
Body weight (kg)								
Day 0	7.02	6.69	7.04	6.73	0.23	0.23	0.62	0.36
Day 7	7.56	7.59	7.78	7.23	0.29	0.89	0.81	0.18
Day 14	9.40	9.81	9.90	9.48	0.36	0.53	0.78	0.43
ADG (g)								
Days 0–7	81	141	101	75	20	0.77	0.03	0.36
Days 7–14	252	317	303	321	21	0.02	0.83	0.55
Days 0–14	166	223	202	198	17	0.12	0.28	0.88
ADFI (g)								
Days 0–7	168	166	168	161	16	0.89	0.97	0.76
Days 7–14	302	335	374	370	24	0.04	0.23	0.90
Days 0–14	234	250	269	260	13	0.08	0.39	0.64
Gain:feed								
Days 0–7	0.464	0.729	0.498	0.437	0.105	0.98	0.07	0.69
Days 7–14	0.933	0.980	0.820	0.865	0.094	0.45	0.26	0.74
Days 0–14	0.753	0.891	0.747	0.764	0.057	0.98	0.08	0.84
Fecal consistency score^2)^								
Day 0	1.06^z^	1.00^y^	1.06^y^	1.15^y^	0.03	0.46	0.08	0.22
Day 7	1.94^x^	1.53^x^	1.53^x^	1.81^x^	0.07	0.14	0.42	0.17
Day 14	1.52^y^	1.31^x^	1.19^y^	1.26^y^	0.06	0.03	0.54	0.65
Overall^3)^	1.51	1.28	1.26	1.41	0.06	0.03	0.45	0.10

NC, negative control; PC, positive control; EF, *Enterococcus faecium*; BP, bacteriophage; ST, *Salmonella typhimurium*; EC, *Escherichia coli*; SEM, standard error of the mean; ADG, average daily gain; ADFI, average daily feed intake.

1) Weanling pigs were fed the basal diet supplemented with none (NC), 3,000 mg ZnO/kg (PC), or 1×10^10^ colony-forming units of EF plus BP (EF-BP) lytic to ST [1×10^8^ plaque-forming units (PFU)/kg] or lytic to each of enterotoxigenic EC F4, F5, and F18 types (EC; 1×10^6^ PFU each/kg) for 14 days. Data are means of four replicates of eight piglets.

2) Fecal consistency was scored subjectively: 1, normal firm feces; 2, soft feces; 3, diarrhea.

3) The p-values for the day and day × treatment interaction were less than 0.01 and 0.36, respectively, with SEM = 0.05 and 0.11, respectively).

x–z Means with no common superscript within a column differ (SEM = 0.11; p<0.05).
